# Series: Practical guidance to qualitative research. Part 7: Qualitative evidence synthesis for emerging themes in primary care research: Scoping review, meta-ethnography and rapid realist review

**DOI:** 10.1080/13814788.2023.2274467

**Published:** 2023-10-30

**Authors:** Albine Moser, Irene Korstjens

**Affiliations:** a Faculty of Health Care, Research Centre Autonomy and Participation of Chronically Ill People, Zuyd University of Applied Sciences, Heerlen, The Netherlands; b Faculty of Health, Medicine and Life Sciences, Department of Family Medicine and Department of Health Service Research, Maastricht University, Maastricht, The Netherlands; c Faculty of Health Care, Research Centre for Midwifery Science, Zuyd University of Applied Sciences, Maastricht, The Netherlands

**Keywords:** Primary care, qualitative synthesis, scoping review, meta-ethnography, rapid realist review

## Abstract

This article, the seventh in a series aiming to provide practical guidance for qualitative research in primary care, introduces qualitative synthesis research for addressing health themes in primary care research. Qualitative synthesis combines rigorous processes and authorial judgement to present the collective meaning of research outputs; the findings of qualitative studies – and sometimes mixed-methods and quantitative research – are pooled. We describe three exemplary designs: the scoping review, the meta-ethnography and the rapid realist review. Scoping reviews aim to provide an overview of the evidence/knowledge or to answer questions regarding the nature and diversity of the evidence/knowledge available. Meta-ethnographies intend to systematically compare data from primary qualitative studies to identify and develop new overarching concepts, theories, and models. Rapid realist reviews aim to provide a knowledge synthesis by looking at complex questions while responding to time-sensitive and emerging issues. It addresses the question, ‘what works, for whom, in what circumstances, and how?’

We discuss these three designs’ context, what, why, when and how. We provide examples of published studies and sources for further reading, including manuals and guidelines for conducting and reporting these studies. Finally, we discuss attention points for the research team concerning the involvement of necessary experts and stakeholders and choices to be made during the research process.

## Introduction

KEY MESSAGES

- Qualitative synthesis combines rigorous processes and authorial judgement to present the collective meaning of research outputs; the findings of qualitative studies – and sometimes mixed-methods and quantitative research – are pooled.
- Scoping reviews aim to provide an overview of the evidence/knowledge or to answer questions regarding the nature and diversity of the evidence/knowledge available.
- Meta-ethnographies intend to systematically compare data from primary qualitative studies to identify and develop new overarching concepts, theories, and models.
- Rapid realist reviews aim to provide a knowledge synthesis by looking at complex questions while responding to time-sensitive and emerging issues: ‘what works, for whom, in what circumstances, and how?’

Qualitative research is specific to a particular context, time and group of participants. During our supervisory work, we noticed that qualitative research tends to evoke many questions and challenges. A frequently asked question was: how can we come from context-rich local qualitative findings to synthesised qualitative findings across contexts? This article, the seventh in a series aiming to provide practical guidance for qualitative research [1], introduces qualitative synthesis for addressing emerging health themes in primary care research. Qualitative synthesis combines rigorous processes and authorial judgement to present the collective meaning of research outputs; the findings of qualitative studies – and sometimes mixed-methods and quantitative research – are pooled [2].

### Challenges in primary care practice

In addition to the challenges, we described in parts 5 and 6 of this Series [3,4] primary care faces challenges in providing evidence-informed care. Practitioners are often familiar with quantitative primary studies, typically reviewed in systematic reviews and meta-analyses. However, they also want to base their decisions on a deeper understanding of their patients’ characteristics and life circumstances. In qualitative studies, ‘thick description’ of natural contexts in which individuals and groups function supports practitioners to consider whether and how the findings can be transferred to their contexts [5].

Finally, for example, during the COVID-19 pandemic, we learned that the traditional ways of generating evidence, such as lengthy systematic reviews, must be complemented with rapid reviews [6]. When researchers tailor the scope and purpose of a rapid review to the evidence request of primary care [7], they can generate the necessary context-sensitive evidence within a short period [8].

### Target audience and content of this article

We regard this article as an introduction to qualitative synthesis. It might be a first acquaintance for researchers - with some experience in qualitative research - who are interested in qualitative synthesis, and for general practitioners who will increasingly read qualitative synthesis articles. We address possible questions about the context and what, why, when and how of these synthesis approaches and their main practical and methodological challenges. We provide examples of published qualitative synthesis studies in primary care and other healthcare domains and sources for further reading, including manuals and guidelines for conducting and reporting these studies.

### Qualitative synthesis

To date, many qualitative synthesis methods exist. Qualitative synthesis includes review types that utilise qualitative approaches to synthesis; predominantly qualitative methods, mixed methods approaches with a qualitative orientation (qualitatising) and mixed methods approaches that handle quantitative and qualitative data equally [9].

Different synthesis methods vary in their respective balance about descriptive and interpretive findings [9]. Essentially, description asks the question ‘What does the data say?’ In contrast, interpretation addresses the more subjective question ‘What does the data mean?’ [9] A descriptive qualitative synthesis might provide an overview or when concepts or themes are well defined in primary research. A more interpretive qualitative synthesis is appropriate for generating concepts, models and frameworks and further developing theory from primary research.

### Essential manuals and guidelines for qualitative synthesis

Qualitative synthesis that draws together evidence regarding complex phenomena requires nuanced, complex thought from readers. Therefore, criteria for rigour in qualitative studies, such as declarations of the researchers’ ‘stance’, researchers’ reflectivity, transparency and triangulation, also apply to qualitative synthesis [2].

During the last decades, Cochrane and Joanna Briggs Institute (JBI) included chapters on qualitative synthesis and scoping reviews in their handbooks [10–12]. Moreover, the Cochrane Qualitative and Implementation Methods Group (QIMG) website provides links to additional supporting series of articles (methods.cochrane.org/qi) (Box 2).

The quantity of the published studies increased and guidelines for conducting and reporting these reviews emerged. One generic reporting tool is available for qualitative synthesis, ‘Enhancing transparency in reporting the synthesis of qualitative research’ (ENTREQ), 21 items within five domains: introduction, methods and methodology (domains 1 and 2), literature search and selection (domain 3), appraisal (domain 4), synthesis of findings (domain 5) [13]. The GRADE-CERQual is an approach for assessing how much confidence to place in the findings of a qualitative synthesis. The overall assessment of confidence is made based on an assessment of four components: methodological limitations, coherence, adequacy, and relevance [14].

Guidelines for reporting specific qualitative synthesis reviews are, for example, PRISMA-ScR for scoping reviews [15], eMERGe for meta-ethnography [16] and PRISMA-RR for rapid reviews and RAMESES II for realist reviews [16–18].

We believe that researchers should use methodological manuals and guidelines in carrying out and publishing a qualitative synthesis.

### Selection of a qualitative synthesis method

Unfortunately, there is no clear-cut algorithm that leads to the choice of a qualitative synthesis method. However, the RETREAT criteria can help a research team select [19]. The RETREAT framework provides seven key considerations that research teams should systematically work through when selecting and planning a qualitative synthesis (Box 1). These are review question – epistemology – time/timescale – resources – expertise – audience and purpose – type of data [19]. Rather than prescribing detailed methodological guidance on individual methods, it seeks to help navigate through various methodology choices. Using the RETREAT considerations, a guide clarifies the factors to consider when selecting a method [9].

In addition, we provide a library of available quality synthesis methods as a resource in this article. Appendix A displays quality synthesis methods using qualitative primary studies. Appendix B displays synthesis methods with primary qualitative and quantitative studies. Appendix C provides an overview of rapid qualitative synthesis methods. Lastly, Appendix D displays qualitative synthesis methods with meta-studies.

### Qualitative synthesis methods discussed

Out of many qualitative synthesis methods, we introduce three exemplary methods, which became common in primary care research: the scoping review, the meta-ethnography and the rapid realist review. Although these methods share characteristics such as the systematic and iterative approach, they differ in, for example, research questions, type of data, type of analysis and timeframe.

For the broad audience in primary care, we chose the scoping review because it can summarise/map data to describe what is known on a relevant topic with a wider conceptual range and focus in contrast to the narrow research questions and parameters examined through traditional systematic reviews.

We choose meta-ethnography because it can support developing a model, framework or theory. Primary care by nature is interprofessional (e.g. general practitioners, nurses, midwives, occupational therapists, speech and language pathologists, physical therapists etc.) and although some primary care professions do not have a long-standing research tradition, they still need to develop conceptual models, frameworks or theories.

Rapid realist reviews offer accelerated synthesis through a streamlined, timely and cost-effective approach directed and guided by stakeholders. We chose this method because recently, the COVID-19 pandemic demanded rapid reviews approaches to address urgent research questions.

Box 1 RETREAT Key considerations when selecting and planning a review, based on Noyes et al. [53]^a^

**Table:** 

Review question	Consider the complexity of the review question. Which elements contribute most to complexity of the phenomenon (e.g., the population, the health problem, the intervention or the context)? Which elements should be prioritised as the essential point for attention?
Epistemology (theory of knowledge)	Consider the philosophical foundations of the primary studies. Would it be appropriate to choose a method such as thematic synthesis so that it is less reliant on epistemological considerations? Would it be appropriate to choose a synthesis method that fits the philosophical foundations of primary studies, such as formal Grounded Theory?
Timeframe	Consider what type of qualitative evidence synthesis will be feasible and manageable within the timeframe available. Does the timeframe allow to carry out a full-blown synthesis? Is the timescale suitable for a rapid synthesis?
Resources	Consider whether the ambition of the review matches the available resources. Will the extent of the scope and the sampling approach of the review need to be limited?
Expertise	Consider access to expertise within the review team and among a wider group of advisors. Does the available expertise support the qualitative evidence synthesis approach chosen?
Audience and purpose	Consider the intended audience and purpose of the review. Does the approach to question formulation, the scope of the review and the intended outputs meet their needs?
Type of data	Consider the type of data present in typical studies for inclusion. How conceptually rich and contextually thick are potential studies in their detail?

^a^ Further explanations of these considerations in Booth et al. and Flemming et al. [19,54].

## Scoping review

### Context

Emerging health issues in primary care require synthesising evidence from quantitative and qualitative research and other sources to identify gaps in the current research and highlight areas requiring further inquiry. Scoping reviews are increasingly used in primary care research because they support addressing broad research questions and mapping evidence/knowledge from various sources [20]. Depending on the research question and available evidence/knowledge, it is possible to undertake a scoping review of only qualitative studies. Scoping reviews can provide overviews to a broad audience for primary care, including primary care services, policymakers, patient groups, and researchers.

The following exemplary articles include published scoping review protocols and scoping reviews:

- Identifying patients with psychosocial problems in general practice: a scoping review protocol [21].
- COVID-19’s impact on primary care and related mitigation strategies: A scoping review [22].
- Care ethics framework for midwifery practice: A scoping review [23].

### What?

Scoping reviews aim to provide an overview of the evidence/knowledge or to answer questions regarding the nature and diversity of the evidence/knowledge available [12]. The researchers aim to determine what kind of evidence/knowledge (quantitative and/or qualitative) exists and to represent this evidence by mapping or charting the data [12]. A scoping review generally does not assess methodological limitations or risk of bias of the evidence, except when the nature of the scoping review aim requires this [12,24], for example, when the objective is to explore the characteristics and methodological quality of knowledge synthesis approaches [15]. The results can generate recommendations or implications for primary research or systematic reviews [25].

### Why and when?

The three most common reasons for a scoping review are to explore the breadth or extent of the literature, to map and summarise the evidence, and to inform future research [26]. The purposes are to identify the types of available evidence/knowledge in a field, clarify key concepts or definitions in the literature, examine how research is conducted on a specific topic or field, identify key characteristics or factors related to a concept, act as a precursor to a systematic review, and identify and analyse evidence/knowledge gaps [27].

### How?

The original methodology for conducting scoping reviews [28] was expanded [29], and enhanced in the JBI framework [12]. The JBI manual [12] guides initiating, developing, undertaking and reporting the review (including an a priori review protocol) and is congruent with the extension for Scoping Reviews of the Preferred Reporting Items for Systematic Reviews Statement – the PRISMA-ScR [15]. Throughout the scoping review process, the researchers consult information scientists, stakeholders and/or content experts, including in the prioritisation, planning, execution and dissemination. The JBI method consists of nine steps in an iterative process:

*Defining and aligning the objective/s and question/s:* The objective may be broad and guides the review scope. The question incorporates the PCC elements: Population, Concept, and Context. There is no need for explicit outcomes, interventions or phenomena of interest but these elements may be implicit in the concept (C).

*Developing and aligning the inclusion criteria with the objective/s and question/s:* The inclusion criteria detail the characteristics of the population (P) that are important for the question. The core concept (C) elements guide the scope and breadth of the inquiry. The context (C) may include cultural factors (such as geographic location and/or specific social, cultural, or gender-based interests) or settings (such as primary care or community). The ‘source’ of information can include any existing literature, such as primary research studies, systematic reviews, meta-analyses, letters, guidelines, websites, blogs. The source can be ‘open’ to include all types of evidence or limited to specific types of evidence/knowledge.

*Describing the planned approach to evidence/knowledge searching, selection, data extraction, and presentation of the evidence/knowledge:* An a priori protocol allows for transparency of the process.

*Searching for the evidence/knowledge:* The iterative search strategy is as comprehensive as possible within the constraints of time and resources to identify published and unpublished (gray or difficult-to-locate literature) primary sources of evidence/knowledge and reviews. The entire search strategy and results are transparent and auditable.

*Selecting the evidence/knowledge:* Two or more researchers perform the source selection, independently, preferably after pilot testing. They provide a description and a flowchart of the process, details of included and excluded sources and the reasons why.

*Extracting the evidence/knowledge:* The iterative data extraction process, also called ‘data charting,’ provides a logical and descriptive summary of the results aligns with the objective/s and question/s. JBI offers a template data extraction form (‘charting table’) in their Appendix 11.1 [12]. The researchers develop and pilot the charting table at the protocol stage and continually update it.

*Analysis of the evidence/knowledge:* Mostly, there are simple frequency counts of concepts, populations, characteristics or other fields of data, sometimes more in-depth analyses. For example, descriptive qualitative content analysis would include basic coding of data (e.g. coding and classifying interventions, strategies and behaviours to a behavioural change model or theory). Principles of framework synthesis may also be useful to chart and sort data against an a priori identified framework. Qualitative content analysis in scoping reviews is generally descriptive.

*Presentation of the results:* The researchers present the results as a map in a diagrammatic or tabular form, and/or in a descriptive format that aligns with the objective/s and scope of the review. The PCC elements may be helpful.

*Summarising the evidence/knowledge in relation to the purpose of the review, making conclusions and noting any implications of the findings:* The researchers discuss the results in the context of current literature, practice and policy and the conclusions match the review objective/s and question/s. They provide clear, specific implications for future research based on the gaps in evidence/knowledge they identified. Any implications for practice align to results that can be used to inform practice. However, implications for practice may not be possible as no assessment of methodological quality and interpretative synthesis takes place.

## Meta-ethnography

### Context

Within health care and primary care, classic systematic reviews on the effects of interventions and health technology assessment prevail but this evidence might not be sufficient. There is an increasing interest in the experiences and perspectives of patients, family carers and professionals to understand the contextual variations that influence interventions and to promote the patient-centeredness of interventions and health technology assessments [10,30].

The following exemplary articles include published empirical studies using meta-ethnography:

- Experiences and perceptions of nutritional health and wellbeing amongst food insecure women in Europe: A qualitative meta-ethnography [31].
- Changing dynamics of caregiving: a meta-ethnography study of informal caregivers’ experiences with older immigrant family members in Europe [32].
- Obstacles to adherence in living with type-2 diabetes: an international qualitative study using meta-ethnography [EUROBSTACLE) [33].

### What

The goal of meta-ethnography is conceptual or theoretical understanding of a particular phenomenon [34,35]. Meta-ethnography uses primary qualitative research that provides an account of a particular social phenomenon, setting or community through thick description of experiences, perceptions, behaviours and practices. A meta-ethnographic approach enables a review of only qualitative studies using multiple designs [34]. Meta-ethnography is an interpretative synthesis, with concepts and an explanatory framework or theory emerging through induction [36]. A meta-ethnography always re-interprets the existing data [35].

### Why and when?

Decisions of policymakers and primary care professionals require the best evidence that is available. The best evidence is ‘all’ the evidence [37]. Meta-ethnography supports that the patient’s experiences and perspectives are fully represented in the evidence base of quantitative systematic reviews of clinical effectiveness, health technology assessments and guidelines [30,38].

Meta-ethnography can refine or revise understanding of a phenomenon. First, it can generate models and theories or provide a historical overview of concepts or theories. Second, it can increase the relevance of findings from single qualitative studies for a broader context, identify directions for future research, and inform when no new conceptual development in a field occurs. Lastly, it can inform the design of complex interventions and enhance the interpretation of systematic reviews of intervention effectiveness and health technology assessments [39].

### How?

A meta-ethnographic study consists of seven, sometimes parallel, steps in an iterative process [34,35].

*Getting started:* The researchers identify an area of interest. They consider whether the topic needs a synthesis and whether a qualitative synthesis and the meta-ethnographic approach fits the research question. To add rigour to the review, they establish a team of researchers with different backgrounds and the key skills to conduct the meta-ethnography [36].

*Deciding what is relevant to the initial interest:* The following activities are: a) defining the focus of the synthesis, b) locating relevant studies, c) making decisions to include studies and d) performing a quality assessment of the included studies.

*Reading the studies:* The synthesis process begins with repeatedly reading the studies and familiarising them with the content and detail of key concepts of the included studies. Consequently, the researchers extract the ‘raw data’, the first and second order constructs, from the primary qualitative studies. First-order constructs are participants’ quotes representing the primary data reported in each qualitative study. Second-order constructs are the themes and concepts representing the primary study authors’ interpretation of the data. Third-order constructs are the review authors’ higher interpretation from the analysis of first- and second-order constructs (see next two steps).

*Determining how the studies are related:* The researchers analyse the relationships between the key concepts from the different studies. They look across the studies for common and recurring concepts that explain and do not only describe the data but also examine the contextual data of each study. They define new concepts to encompass all the relevant categories (from first- and second-order concepts).

*Translating the studies into one another:* The researchers compare each concept from each study with the concepts in all the other papers to identify similarities and differences resulting in new concepts. The researchers organise the new concepts into conceptual categories. Then they develop them further into higher third-order constructs. Meta-ethnographic analysis should develop concepts, themes or models that help to understand an experience.

*Synthesising the translations:* The researchers develop a framework or theory within two parallel analytical processes. First, the primary studies that are sufficiently similar in their focus allow for a reciprocal translation synthesis. Second, the primary study findings that contradict each other require a refutational translation synthesis. Finally, the researchers create a line of argument synthesis by constantly comparing the third-order concepts and developing the framework or theory. These are the findings from ethnography.

*Expressing the synthesis:* The researchers write down the framework or theory following the eMERGe reporting guidance [16].

## Rapid realist review

### Context

In primary care, emerging health issues and the implementation of complex interventions require rapid access to high-quality evidence, promptly and within short timeframes [40]. The value of rapid realist reviews lies in providing context-sensitive understanding of how health programmes or interventions work in different settings and under other circumstances [41]. Their target audiences include all kinds of local stakeholders: policymakers, healthcare institutions, primary care professionals, and patient organisations.

The following exemplary articles include published rapid realist reviews:

- Understanding the implementation of interventions to improve the management of frailty in primary care: a rapid realist review [7].
- How do community-based dementia-friendly initiatives work for people with dementia and their caregivers, and why? A rapid realist review [42].
- Care Planning: what works, for whom, and in what circumstances? A rapid realist review [43].

### What?

Rapid realist reviews are based on the realist review methodology and rely heavily on qualitative evidence [44]. They are theory-driven and aim to understand whether an intervention works in a specific context and to understand the processes leading to success or otherwise. The underlying principles are to describe a programme theory explaining the links between interventions (I), contexts (C), mechanisms (M) and outcomes (O) [44]. These reviews have an explanatory focus.

### Why and when?

A rapid realist review generates evidence that suggests that an inevitable intervention is more or less likely to work [41]. It can inform guideline recommendations in urgent and emergent health situations [41,45] as it often provides knowledge synthesis within 1 to 6 months [26].

Rapid realist reviews are appropriate for stakeholders who seek timely evidence syntheses that highlight possible interventions (I) that could be implemented within a specific context (C) that, in turn, interact with various mechanisms (M) and produce outcomes (O) of interest. Or, more simply, stakeholders want to explore how, why, for whom, and in what circumstances an intervention works. The value of a rapid realist review for primary care is that it prioritises deep understanding of contextual aspects and can support developing local health policy, health service programmes and interventions [8].

### How?

Rapid realist reviews are responsive to limited time and resources and can integrate empirical, theoretical and practical knowledge [41]. The researchers are responsible for taking design decisions and practical steps to reduce the timeframe [8]. Furthermore, they explicitly engage stakeholders (content experts and end-users) to define the specific research questions of interest, identify literature and knowledge gaps and streamline the review process [46,47]. It is an iterative process. Saul provides a guideline to conduct a rapid realist review in 10 steps [41]:

*Development of the project scope:* The researchers and stakeholders clarify the content area of interest. This is critical for a feasible review process, regardless of the desired timeline.

*Development of specific research questions:* Researchers and stakeholders discuss the questions that are most important to stakeholders and they might refine these questions to enable collecting sufficient evidence.

*Identification of how the findings and recommendations will be used:* The formulation of a purpose statement helps identify how the stakeholders will use the review findings. The use of review products is a key element in these reviews.

*Development of search terms:* The researchers and stakeholders identify terms likely to be relevant to the project scope, purpose, and research questions.

*Identifying articles and documents for inclusion in the review:* The stakeholders and content experts drawn from the pool of stakeholders generate an initial list of published research articles and grey literature documents. Content experts have experience in the field and can fill gaps in the literature by representing experiential and professional knowledge. Next, adding more search terms supports iteratively expanding the list for inclusion.

*Quality review:* The researchers narrow down the most relevant search terms. Simultaneously, stakeholders and content experts further identify published and grey key documents to accelerate the search process. A search will not be comprehensive. This procedure, combined with the validation step (see below), helps to ensure that no significant literature will be missing.

*Extraction of data from the literature:* The researchers use an extraction template to select the data for answering the research questions. They analyse the findings to build a format that addresses the agreed focus and scope of the review. Data is extracted and grouped to identify context-mechanism-outcomes and emerging patterns. Consequently, context-mechanisms-outcomes and emerging patterns are synthesised and programme theories are proposed. They are tested and refined with additional evidence.

*Validation of findings with content experts:* The researchers generate the programme theory and experts with experience in the field review it.

*Synthesis of the findings in a final report:* The researchers write the final report in a way that meets the needs of the stakeholders.

*Dissemination of results:* The researchers work with the stakeholders to apply the findings. This is possible because the findings focus on interventions that can be implemented within the stakeholders’ context. A programme theory is presented as a way to understand how changes in context may interact with mechanisms to produce outcomes of interest. To date, there is a published protocol for extending rapid review to the Preferred Reporting Items for Systematic Reviews and Meta-Analysis (PRISMA) [17]. Researchers might consult the RAMESES II reporting standard [18].

## Challenges and strategies in qualitative synthesis research

We provide strategies for addressing the main practical and methodological challenges in qualitative synthesis research.

### Practical attention points for the research team

*Expertise:* The composition of the review team is critical for qualitative synthesis [41]. The review team minimally comprised of experienced reviewers, information specialists, analysis specialists (qualitative analysis experts and/or statisticians) and content experts. Depending on the synthesis method used other stakeholders such as policymakers, patient representatives and practitioners might be essential members too.

All members should be well-informed about the review methodology. The principal investigator must be able to value and connect their different types of knowledge. Spending time formulating a clear sense of the scope and purpose at the start of the review process supports further refining the research question [48].

*Working with stakeholders and content experts*: Qualitative synthesis often involves stakeholders and content experts in different stages with different levels of involvement. It is best practice to engage with key stakeholders, content experts and patient and public representatives - from inception to dissemination - to ensure that the review is developed with multiple actor perspectives and that the findings are grounded in reality [49]. This might be challenging because of turnover, workload or unfamiliarity with qualitative synthesis. The principal investigator must ensure continuous involvement (including approaching new stakeholders or content experts) and adequately plan budgeting or compensation.

*The following exemplary articles included are published:* Qualitative synthesis uses well-considered sampling strategies rather than exhaustive inclusion. In our three examples, scoping reviews use purposive sampling [12], meta-ethnography starts with purposive sampling and maximum variation sampling followed by theoretical sampling and rapid realist reviews mostly use convenience and snowball sampling [41,50]. Closely involved stakeholders and content experts who know ‘their sources’ can provide valuable grey literature. The principal investigator should have a broad view of what counts as ‘evidence/knowledge.’

### Methodological challenges

*Research questions:* In qualitative synthesis, research questions must be broad and open to unexpected findings that might ask for fine-tuning or additional questions within the review’s scope [5]. We recommend that the researchers register and publish the review protocol and, later in the publication, should report methodological modifications [12]. For registration in the PROSPERO database (https://www.crd.york.ac.uk/prospero/), qualitative synthesis and rapid reviews with health-related outcomes are eligible but not yet scoping reviews. Registration is possible with the Open Science Framework (https://osf.io/) or Figshare (https://figshare.com/) and some journals, such as the JBI Evidence Synthesis, publish scoping review protocols.

*Search strategies:* Literature searching would be comprehensive or iterative appropriate to the review’s focus. Challenges are, for example, lacking detailed thesaurus terms for qualitative research or for specific qualitative methods [11], non-informative titles and abstracts, diffuse terminology, and poor indexing [10]. This may require additional search strategies such as reference checking, citation checking, hand searching and contact with authors or subject experts. Researchers should provide detailed reports of all searches, including the searches’ dates. The input of a research librarian or information scientist can be invaluable in designing and refining the search.

*Quality assessment:* The issue of whether to undertake and how to use a quality assessment of the methodological strengths and limitations of primary qualitative and quantitative studies for potential inclusion remains contentious, with often divided opinions [49]. Although quality assessment is rare in scoping and rapid reviews, researchers should generally be transparent about the process [51] and report some sort of quality assessment [49]. A standard tool to do this is The Critical Appraisal Skills Programme (CASP) checklist for qualitative studies covering 3 broad issues: A. Are the results of the study valid? B. What are the results? C. Will the results help locally? [52].

At present, there is no thoroughly evaluated and tested tool to assess the rigour of a quality synthesis report [49], although several guidelines for reporting exist, such as ENTREQ, PRISMA-ScR, eMerge, PRISMA-RR, RAMESES II, and the GRADE-CERQual tool for assessing how much confidence to place in the findings of a qualitative synthesis (Box 2).

*Data extraction, aggregation and synthesis:* Most qualitative synthesis guidelines recommend that two or more researchers perform the data extraction, aggregation or synthesis in an iterative process [11,12,53]. Review teams will need regular meetings to discuss and further interrogate the evidence and achieve a shared understanding [10,34]. Different types of qualitative synthesis provide other specific recommendations for this process. If not, the principal investigator should determine the role of each member of the research team members in data extraction, aggregation and synthesis in the review protocol.

## Further reading

We hope this seventh introductory paper in our Series provides a basic understanding of qualitative synthesis research for general practitioners and researchers facing health themes in primary care. A deeper understanding is necessary to apply these approaches in research projects. Therefore, we provide sources for further reading, including manuals and guidelines for conducting and reporting these studies (Box 2).

Box 2 Sources for further reading.

**Table:** 

***Qualitative evidence synthesis*** Cochrane Qualitative and Implementation Methods Group (QIMG) website (methods.cochrane.org/qi): links to Cochrane handbook, overview paper, webinars, QIMG guidance series in J Clin Epidemiol. 2017-2018, and Applying GRADE-CERQual to qualitative evidence synthesis series in Implementation Science 2018. Lewin S, Bohren M, Rashidian A, et al. Applying GRADE-CERQual to qualitative evidence synthesis findings—paper 2: How to make an overall CERQual assessment of confidence and create a summary of qualitative findings table. Implementation Science 2018; 13: 10. Available from https://doi.org/10.1186/s13012-017-0689-2 Lockwood C, Porritt K, Munn Z, et al. Chapter 2: Systematic reviews of qualitative evidence. In: Aromataris E, Munn Z (Editors). JBI Manual for Evidence Synthesis. JBI, 2020. Available from: https://synthesismanual.jbi.global. https://doi.org/10.46658/JBIMES-20-03. Noyes J, Booth A, Cargo M, et al. Chapter 21: Qualitative evidence. In: Higgins JPT, Thomas J, Chandler J, Cumpston M, Li T, Page MJ, Welch VA (editors). Cochrane Handbook for Systematic Reviews of Interventions version 6.3 (updated February 2022). Cochrane, 2022. Available from www.training.cochrane.org/handbook. Tong A, Flemming K, McInnes E, et al. Enhancing transparency in reporting the synthesis of qualitative research: ENTREQ. BMC Medical Research Methodology 2012;12:181. ***Scoping review***: Arksey H, O’Malley L. Scoping studies: towards a methodological framework. Int J Soc Res Methodol. 2005;8:19-32. Levac D, Colquhoun H, O’Brien KK. Scoping studies: advancing the methodology. Implementation Sci. 2010;5:1-9. Peters MDJ, Godfrey C, McInerney P, et al. Chapter 11: Scoping Reviews (2020 version). In: Aromataris E, Munn Z (Editors). JBI Manual for Evidence Synthesis, JBI, 2020. Available from https://synthesismanual.jbi.global. https://doi.org/10.46658/JBIMES-20-12 Tricco, A.C., Lillie, E., Zarin, W., et al., 2018. PRISMA extension for scoping reviews (PRISMA-ScR): checklist and explanation. Annals of Internal Medicine, 2018;169(7):467-473 ***Meta-ethnography*** Campbell R, Pound P, Morgan M, et al. Evaluating meta-ethnography: systematic analysis and synthesis of qualitative research. Health Technol Assess. 2011;15:1-164. France E, Cunningham M, Ring N, et al. Improving reporting of Meta-Ethnography: the eMERGe Reporting Guidance. BMC Medical Research Meth. 2019;19:25. Noblit GW, Hare RD: Meta-Ethnography: Synthesizing Qualitative Studies 1988. London. Sattar R, Lawton R, Panagioti M. et al. Meta-ethnography in healthcare research: a guide to using a meta-ethnographic approach for literature synthesis. BMC Health Serv Res. 2021;21:50. ***Rapid realist review*** Garritty C, Gartlehner G, Nussbaumer-Streit B, et al. Cochrane Rapid Reviews Methods Group offers evidence-informed guidance to conduct rapid reviews. J Clin Epidemiol. 2021;130:13-22. Saul, J.E., Willis, C.D., Bitz, J. et al. A time-responsive tool for informing policy making: rapid realist review. Implementation Sci. 2013;8:103. Stevens A, Garritty C, Hersi M, et al. Developing PRISMA-RR, a reporting guideline for rapid reviews of primary studies (Protocol). The Equator Network; 2018:12. Available from: https://www.equator-network.org/wp-content/uploads/2018/02/PRISMA-RR-protocol.pdf. Tricco AC, Antony J, Zarin W, et al. A scoping review of rapid review methods. BMC Med. 2015;13:224. Wong G, Westhorp G, Manzano A, et al. RAMESES II reporting standards for realist evaluations. BMC Med. 2016;14(1):96.

## Appendices: Library qualitative synthesis designs

- Appendix A Library qualitative synthesis with primary qualitative studies
- Appendix B Library qualitative synthesis methods with primary qualitative and quantitative studies
- Appendix C Library rapid qualitative synthesis methods
- Appendix D Library qualitative synthesis methods with meta-studies

## Appendix A: Library qualitative synthesis with primary qualitative studies

**Table:**

Design	Reference
Cochrane qualitative evidence review	Noyes J, Booth A, Cargo M, et al. Chapter 21: Qualitative evidence. In: Higgins JPT, Thomas J, Chandler J, Cumpston M, Li T, Page MJ, Welch VA (editors). Cochrane Handbook for Systematic Reviews of Interventions version 6.3 (updated February 2022). Cochrane, 2022. Available from: Chapter 21: Qualitative evidence | Cochrane Training
Concept analysis	Walker LO, Avant KC. Strategies for Theory Construction in Nursing. 4th ed. Pearson Prentice Hall: Upper Saddle River; 2005.
Content analysis: content	Evans D, Fitzgerald M. Reasons for physically restraining patients and residents: a systematic review and content analysis. Int J Nurs Stud. 2002;39:739–743.
Content analysis: frequencies of categories	Stemler S. An overview of content analysis. Pract Asses Res Eval. 2001;7:17.
Critical review	Grant MJ, Booth A. A typology of reviews: An analysis of 14 review types and associated methodologies. Health Info Lib J. 2009:26,91–108.
Evidence summary	Khangura S, Konnyu K, Cushman R, et al. Evidence summaries: The evolution of a rapid review approach. Syst Rev. 2012;1:10.
Evidence synthesis (including 12 synthesis approaches)	Joanna Briggs Institute Aromataris E, Munn Z (Editors). JBI Manual for Evidence Synthesis. JBI, 2020. Available from: https://synthesismanual.jbi.global
Expert opinion review	McArthur A, Klugarova J, Yan H, et al. Innovations in the systematic review of text and opinion. Int J Evid Based Health. 2015;13:188–195.
Framework synthesis and ‘Best fit’ framework analysis	Pope C, Ziebland S, Mays N. Qualitative research in health care: analysing qualitative data. BMJ 2000; 320:114–116. Oliver S, Rees R, Clarke-Jones L, et al. A multidimensional conceptual framework for analysing public involvement in health services research. HEX 2008;11:72–84. Carroll C, Booth A, Cooper K. A worked example of "best fit" framework synthesis: A systematic review of views concerning the taking of some potential chemopreventive agents. BMC Med Res Methodol. 2011;11:29.
Integrative review	Whittemore R, Knafl K. The integrative review: Updated methodology. J Adv Nurs. 2005; 52: 546–553.
Meta-aggregation	Hannes K, Lookwood C. Pragmatism as the philosophical foundation for the Joanna Briggs meta‐aggregative approach to qualitative evidence synthesis. J Adv Nurs. 2022;67:1632–1642.
Meta-ethnography	Noblit GW, Hare R. Meta-Ethnography: synthesising qualitative studies. London: Sage; 1988. Campbell R. Pound P, Morgan M, et al. Evaluating meta-ethnography: systematic analysis and synthesis of qualitative research. Health Tech Ass. 2011;15:1–164 Available from: Evaluating meta-ethnography: systematic analysis and synthesis of qualitative research (nihr.ac.uk)
Meta-interpretations	Weed M. 'Meta-interpretation’: a method for the interpretive synthesis of qualitative research. FQS. 2005;6:37. Available from: View of "Meta Interpretation": A Method for the Interpretive Synthesis of Qualitative Research | Forum Qualitative Sozialforschung / Forum: Qualitative Social Research (qualitative-research.net)
Meta-narrative	Greenhalg T, Robert G, Macfarlane F, et al. Storylines of research in diffusion of innovation: a meta-narrative approach to systematic review. Soc Sci Med. 2005;61:417–430.
Metasynthesis	Sandelowski M, Docherty S, Emden C. Qualitative metasynthesis: Issues and techniques. Res Nurs Health. 1997;20:365–371.
Meta-synthesis	Fingeld DL. Meta-synthesis: the state-of-the-art so far. Qual Health Res. 2003;13:893–904.
Meta-study	Paterson BL, Thorne SE, Canam C, et al. Meta-Study of qualitative health research. A Practical Guide to Meta-Analysis and Meta-Synthesis. Thousand Oaks: Sage; 2001.
Qualitative cross-case analysis	Miles MB, Huberman AM. Qualitative data analysis: A sourcebook of new methods. 2nd ed. Beverly Hills: Sage; 1994.
Qualitative evidence synthesis	Noyes J, Booth A, Cargo M, et al. Chapter 21: Qualitative evidence. In: Higgins JPT, Thomas J, Chandler J, Cumpston M, Li T, Page MJ, Welch VA (editors). Cochrane Handbook for Systematic Reviews of Interventions version 6.3 (updated February 2022). Cochrane, 2022. Available from: www.training.cochrane.org/handbook.
Qualitative interpretive meta-synthesis	Aguirre RT, Bolton KW. Qualitative interpretive meta-synthesis in social work research: Uncharted territory. J Soc Work. 2014;14:279–294.
Qualitative metasummary	Sandelowski M, Barroso J. Handbook for Synthesising Qualitative Research. New York: Springer Publishing Company; 2007.
Qualitative meta-synthesis	Zimmer L. Qualitative meta-synthesis: a question of dialoguing with texts. J Adv Nurs. 2006;53:311–318.
Qualitative systematic review	Popay J, Rogers A, Williams G. Rationale and standards for systematic review of qualitative literature in health service research. Qual Health Res. 1998;8:341–351 Grant MJ, Booth A. A typology of reviews: an analysis of 14 review types and associated methodologies. Health Infor Lib J. 2009;26:91–108.
Qualitative research synthesis	Savor CH, Savin-Baden M. An introduction to qualitative research synthesis: Managing the information explosion in social science research. London: Routledge; 2010.
State-of-the-art review	Grant MJ, Booth, A. A typology of reviews: An analysis of 14 review types and associated methodologies. Health Infor Lib J. 2009;26,91–108
Thematic synthesis	Thomas J, Harden A. Methods for the thematic synthesis of qualitative research in systematic reviews. BMC Med Res Methodol. 2008;10:45.

## Appendix B: Library Synthesis with primary qualitative and quantitative studies

**Table:**

Design	Reference
Bayresian meta-analysis	Sutton AJ, Abrams KR: Bayesian methods in meta-analysis and evidence synthesis. Statistical Stat Method Med Res. 2001;10:277–303. Roberts KA, Dixon-Woods M, Fitzpatrick R, et al. Factors affecting uptake of childhood immunisation: a Bayesian synthesis of qualitative and quantitative evidence. Lancet. 2002;360:1596–1599.
Critical interpretive synthesis	Dixon-Woods M, Cavers D, Agarwal S, et al. Conducting a critical interpretive synthesis of the literature on access to healthcare by vulnerable groups. BMC Med Res Methodol. 2006;6:35.
Content analysis	Seuring S, Gold, S. Conducting content-analysis based literature reviews in supply chain management. Supply Chain Management. 2012;17:544–555.
Critical interpretist synthesis (CIS)	Flemming K. Synthesis of quantitative and qualitative research: an example using Critical Interpretive Synthesis. J Adv Nurs. 2010;66,201–217.
Cross-case analysis	Yin R. Case study research, design and methods. Applied Social Research Methods Series Vol 5. 3rd ed. Thousand Oaks CA: Sage; 2003.
Cross-design synthesis	Droitcour J, Silberman G, Chelimsky E. Cross-design synthesis: a new form of meta-analysis for combining results from randomised clinical trials and medical-practice databases. Int J Tech Ass Health Care. 1993;9:440–927.
Ecological triangulation	Banning JH. Ecological triangulation: an approach for qualitative meta-synthesis (What works for youth with disabilities project: US Department of Education. 2003.
EPPI-centre outcomes plus views review	Oliver S. Advantages of concurrent preparation and reporting of systematic reviews of quantitative and qualitative evidence. J Royal Soc Med. 2015;108:108–111.
Evidence maps	Katz DL, Williams AL, Girard C, et al. The evidence base for complementary and alternative medicine: methods of evidence mapping with application to CAM. Altern Ther Health Med. 2003;9:22–30.
Metasummary	Sandelowski M, Barroso J, Voils CS. Using qualitative metasummary to synthesise qualitative and quantitative descriptive findings. Res Nurs Health. 2007;30:99–111.
Mixed-methods review *Primary qualitative, quantitative mixed-methods studies	Pluye P, Gagnon MP, Griffiths F, et al. A scoring system for appraising both methods research, and concomitantly appraising qualitative, quantitative and both methods primary studies in both studies reviews. Int J Nurs Stud. 2009;46:529–546.
Narrative summary	Dixon-Woods M, Agarwal S, Jones D, et al. Synthesising qualitative and quantitative evidence: a review of possible methods. J Health Serv Res Policy. 2005;10:45–53.
Qualitative case survey	Yin R, Heald K. Using the case survey method to analyse policy studies. Adm Scie Q. 1975;20:371–381.
Realist review	Pawson R, Greenhalgh T, Harvey G, et al. Realist review--a new method of systematic review designed for complex policy interventions. J Health Serv Res Policy. 2005;10:21–34.
Scoping review	Arksey H, O’Malley L. Scoping studies: towards a methodological framework. Int J Soc Res Methodol. 2005;8:19–32. Levac D, Colquhoun H, O’Brien KK. Scoping studies: advancing the methodology. Implementation Sci. 2010;5:1–9.
Textual narrative synthesis	Lucas PJ, Arai L, Baird, Law C, Roberts HM. Worked examples of alternative methods for the synthesis of qualitative and quantitative research in systematic reviews. BMC Med Res Methodol. 2007,7:4.
Thematic analysis	Mays N, Pope C, Popay J: Systematically reviewing qualitative and quantitative evidence to inform management and policymaking in the health field. J Health Serv Res Policy. 2005;10:6–20.

## Appendix C: Library rapid qualitative synthesis

**Table:**

Rapid evidence assessment	Varker T, Forbes D, Dell L, et al. Rapid evidence assessment: increasing the transparency of an emerging methodology. J Eval Clin Pract. 2015;21:1199–1204.
Rapid review	Tricco AC, Langlois EV, Straus SE (editors). Rapid reviews to strengthen health policy and systems: a practical guide. Geneva: World Health Organisation; 2017. Available from: 9789241512763-eng.pdf (who.int)
Rapid realist review	Saul JE, Willis CD, BitzJ. et al. A time-responsive tool for informing policy making: rapid realist review. Implementation Sci. 8:103.
Rapid scoping review	Tricco AC, Thomas SM, Antony J, et al. Strategies to Prevent or Reduce Gender Bias in Peer Review of Research Grants: A Rapid Scoping Review. PLoS ONE 2017;12(1):e0169718.
Rapid systematic review of reviews	Caird J, Rees R, Kavanagh J, et al. The socioeconomic value of nursing and midwifery: a rapid systematic review of reviews. London, UK, EPPI-Centre, Social Science Research Unit, Institute of Education, University College London: London; 2010. Available from: The socioeconomic value of nursing and midwifery: A rapid systematic review of reviews
Health technology rapid reviews	Harker J, Kleijnen J. What is a rapid review? A methodological exploration of rapid reviews in Health Technology Assessments. Int J Evid Based Healthc. 2012;10:397–410

## Appendix D: Library qualitative synthesis with meta-studies

**Table:**

Cochrane overviews of reviews	Pollock M, Fernandes RM, Becker LA, et al. Chapter V: Overviews of Reviews. In: Higgins JPT, Thomas J, Chandler J, Cumpston M, Li T, Page MJ, Welch VA (editors). Cochrane Handbook for Systematic Reviews of Interventions version 6.3 (updated February 2022). Cochrane, 2022. Available from: www.training.cochrane.org/handbook.
Grounded formal theory *Primary grounded theory studies	Strauss AL, Corbin J. Basics of qualitative research: techniques and procedures for developing Grounded Theory. Thousand Oaks: Sage; 1998. Eaves YD. A synthesis technique for grounded theory data analysis. J Adv Nurs. 2001;35:654-663
Mega-aggregation framework Synthesis	Hendricks L, Eshun-Wilson I, Rohwer, A. A mega-aggregation framework synthesis of the barriers and facilitators to linkage, adherence to ART and retention in care among people living with HIV. Syst Rev. 2021:10, 54.
Mega-ethnography	Toye F, Seers K, Hannink E, et al. A mega-ethnography of eleven qualitative evidence syntheses exploring the experience of living with chronic non-malignant pain. BMC Med Res Methodol. 2017;17:116.
Umbrella review approach *Systematic review of QES	Aromataris E, Fernandez R, Godfrey CM, et al. Summarising systematic reviews: methodological development, conduct and reporting of an umbrella review approach. Int J Evid Based Healthc. 2015;13:132-140.
